# LOCALI: Calibration-Free Systematic Localization Approach for Indoor Positioning

**DOI:** 10.3390/s17061213

**Published:** 2017-05-25

**Authors:** Muhammad Usman Ali, Soojung Hur, Yongwan Park

**Affiliations:** Department of Information and Communication Engineering, Yeungnam University, Gyeongsan 38541, Korea; musmanali@ynu.ac.kr (M.U.A.); sjheo@ynu.ac.kr (S.H.)

**Keywords:** indoor positioning, IPS, ILBS, calibration free, localization, fingerprinting

## Abstract

Recent advancements in indoor positioning systems are based on infrastructure-free solutions, aimed at improving the location accuracy in complex indoor environments without the use of specialized resources. A popular infrastructure-free solution for indoor positioning is a calibration-based positioning, commonly known as fingerprinting. Fingerprinting solutions require extensive and error-free surveys of environments to build radio-map databases, which play a key role in position estimation. Fingerprinting also requires random updates of the database, when there are significant changes in the environment or a decrease in the accuracy. The calibration of the fingerprinting database is a time-consuming and laborious effort that prevents the extensive adoption of this technique. In this paper, we present a systematic LOCALIzation approach, “LOCALI”, for indoor positioning, which does not require a calibration database and extensive updates. The LOCALI exploits the floor plan/wall map of the environment to estimate the target position by generating radio maps by integrating path-losses over certain trajectories in complex indoor environments, where triangulation using time information or the received signal strength level is highly erroneous due to the fading effects caused by multi-path propagation or absorption by environmental elements or varying antenna alignment. Experimental results demonstrate that by using the map information and environmental parameters, a significant level of accuracy in indoor positioning can be achieved. Moreover, this process requires considerably lesser effort compared to the calibration-based techniques.

## 1. Introduction

**Figure 1:**
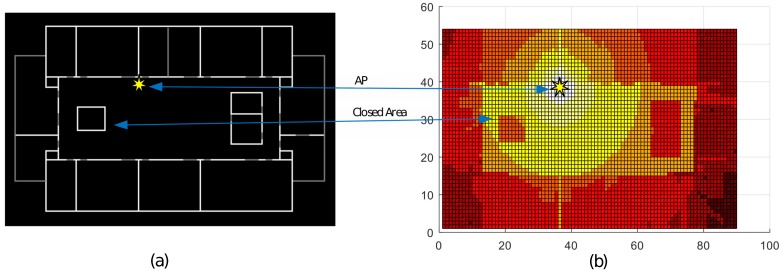
Site wall-map and the corresponding estimated radio-map, clearly showing the change in received signal strength indicator(RSSI) with respect to line-of-sight and non-line-of-sight cases; (**a**) site wall-map and (**b**) RSSI radio-map of an Access Point.

After the successful implementation and deployment of outdoor location based services (OLBS) using global positioning systems (GPSs), indoor positioning systems (IPS) are now popular. GPS signals from the satellites are prone to multi-path effects in indoor environments (the so-called GPS-denied environments) and are unable to provide a certain level of accuracy in location estimation. This drawback renders the GPS unsuccessful for indoor positioning. Indoor location-based services (ILBS) are expected to play an important role in a diverse range of services. The ILBS enables a user to track devices/users in an indoor environment. In recent years, several solutions have been proposed for the ILBS, providing different levels of accuracy, commonly known as "application specific accuracy levels". IPS solutions are categorized mainly into infrastructure-based and infrastructure-free. Infrastructure-based solutions require the pre-installation of special-purpose hardware infrastructure (e.g., Radio-frequency identification (RFID), Radio Sensors, Bluetooth, Ultra Wide Band(UWB), etc.) [1,2,3,4,5] and some of them provide a high level of accuracy. However, these solutions are less attractive due to deployment costs, whereas the infrastructure-free solutions are more attractive due to low costs and ready-to-deploy characteristics. Infrastructure-less solutions commonly utilize the existing Wi-Fi infrastructure [6] or Light-emitting diode (LED). lights [7] or imaging scene structure [8,9] or other resources that are already available in the environment to estimate the target position. As Wi-Fi infrastructures are commonly available in most public-service and private corporate buildings, most of the proposed ILBS solutions exploit Wi-Fi signals for indoor positioning. We can divide these solutions into two major approaches, i.e., calibration-based and calibration-free, also known as database-based/map-free and database-free/map-based solutions, respectively. The indoor environment is a hybrid owing to the co-existence of line-of-sight (LOS) and non-line-of-sight (NLOS) cases. This complexity in the indoor environment restricts the use of trilateration or triangulation techniques for positioning. The time of arrival (TOA) or time difference of arrival (TDOA) [10,11] suffers from multi-path propagation error and also from other issues such as time synchronization problems and short-range timing accuracies, respectively, whereas the angle of arrival (AOA) requires complex hardware for angle calculation. The received signal strength indicator (RSSI) value is a solution for avoiding the time synchronization but is affected by fading due to several reasons e.g., multi-path propagation or absorption for example by humans or varying antenna alignment. The fingerprinting technique offers a good balance between effort and accuracy for many use cases, currently available, for such complex indoor environments, addressing the fading error issue for Wi-Fi-based IPS. It is a calibration-based approach for enabling IPBS in GPS-denied environments; however, it also suffers from the challenging issue of tedious and time-consuming random database calibrations, which restrict its wide adoption. Several solutions have been suggested for reducing the construction and update time of the database [12]. Updates are required, if there are significant changes in the environment. In this work, we present a calibration-free positioning technique, which leverages the map information and provides a robust and efficient method for estimating indoor positioning, without a calibration database. We use the map information to estimate the propagation model for the LOS and NLOS, and construct an RSSI map for each access point (AP), as shown in Figure 1. Using these RSSI heat maps, we then estimate the target location using an overlap technique that employs a much simpler algorithm than the other techniques for position estimation (e.g., trilateration, triangulation and multilateration). Our approach is a step toward a simple and robust technique for calculating the path loss (by absorption and reflection) of the Wi-Fi signals through complex indoor environments for the construction of RSSI maps. Furthermore, it estimates the target location using the RSSI list received at the target location, as a system input, without trilateration or triangulation techniques. In this work, we briefly describe the advantages of our approach, which promotes adaptable, easy, and ready-to-be-deployed indoor location-bases services. Most of the proposed solutions estimate the location by iteration through all of the possible locations, whereas the LOCALI estimates the location directly using the map overlap technique. We use the words sender, transmitter, and AP alternatively in this manuscript.

In Section 2, we discuss some of the problems and challenges in the existing technologies. In Section 3, the proposed approach is discussed; Section 4 discusses the experimental details and results. Section 5 concludes the study.

## 2. Existing Systems

In most of the proposed indoor positioning techniques, target localization is based on the signal propagation time or the received signal strength (RSS) information from multiple transmitters, i.e., Wi-Fi APs. Trilateration and triangulation, which use the propagation time information, undergo an unacceptable accuracy limitation due to certain constraints; the TOA requires a high level of precision in the time synchronization, at both the sender and receiver sides. This becomes more critical, when synchronization is done for the very short ranges of an indoor environment. Specialized directional antennas are required to accurately assess the AOA of the RF signals. The TDOA is used to avoid the time synchronization problem, but it has multi-path propagation delay errors inside the building due to walls or obstructions. Instead of using the vulnerable time information, most studies use the RSSI, a metric that is most commonly used in IPS as a substitute for the time information, with respect to the distance between the sender and receiver. However, the RSSI also suffers from fading due to multi-path, absorption by soft objects and varying antenna alignment in a multi-modal environment [13], and it is almost impossible for the RSSI level to be circular with respect to the distance from the transmitter to the receiver. Hence, we cannot use trilateration directly to estimate the position of the target. Fingerprinting is a calibration-based solution for such trilateration and triangulation problems. In this study, we place emphasis on the fingerprinting technique using the RSSI of the Wi-Fi network [14,15], whereas fingerprinting can also be carried out using other kinds of signal or field strength (e.g., geo-magnetism) [16]. Fingerprinting has two phases: the pre-deployment offline survey training phase and the online location estimation phase. It requires a comprehensive survey of the environment to construct an RSSI database at a certain degree of spatial granularity. This training point granularity is significant with respect to the level of accuracy required from the fingerprinting technique. It is time-consuming to record the RSSI level at each grid point of the environment; it becomes more challenging, when the device orientation, the types of devices used in the survey, and the averaging of the RSSI level due to the time varying characteristics of the RSSI are to be considered. Of late, extensive research efforts have been made to reduce this laboriously time-consuming fingerprinting training phase and several solutions have been proposed [12].

### 2.1. Interpolation-Based Systems

Some of the solutions attempt to reduce the survey time using interpolation techniques with a modified path-loss model at sparse reference points. In [17], the author combines the fingerprint prediction model with a calibration procedure and proposed a new hybrid model that provides a comparable location accuracy using a few RSSI samples to that of the traditional fingerprinting approach. Triangular interpolation and extrapolation (TIX) [18] uses only online Wi-Fi RSS values measured at each AP to obtain a linear mapping between RSS decay and distance, which further helps in estimating client location using the TIX algorithm. Similarly, a signal-distance map (SDM) [19] works on the same principle, whereas the mapping of distance between APs and RSS measurements is obtained by singular value decomposition (SVD).

### 2.2. Crowdsourcing-Based Systems

Systems employ active user inputs or crowdsourcing [20,21,22,23,24,25,26] to build a radio-map database online instead of using the conventional time-consuming task of building a training database through a survey. The Organic indoor Localization OIL [22] incorporates the active user input after training phase to extend the converge and accuracy of fingerprinting based localization at particular areas called varonoi regions. EZ [23] does not require prior map and transmitter location information and resolves the environment, devices and distance parameters by running a genetic algorithm on RSS information reported by a background service running on user mobiles. Walkie-Markie [24] also relaxes the prior information of indoor RF infrastructure and tries to generate maps of the environment using crowd-sourcing. The Wifi-mark estimation in the Walkie-Markie requires a sufficient number of users reporting the trajectory information on at least two sides of an AP, which is nearly impractical from the user’s point of view and the building architecture, with corners and long continuous hallways in which traversal to the other side is not usually possible.

### 2.3. Sensors-Based Systems

Recent research developments in ILBS technology use data from a wide range of sensors, i.e., inertial sensors, accelerometers, gyroscopes, magnetic sensors, etc. to improve indoor positioning and mapping [27] using data fusion techniques [28,29,30,31]. WILL (Wireless Indoor Localization) and unLoc trace user movements with an inertial sensor to generate a map of the environment [32,33].

### 2.4. Model-Based Systems

A model-based indoor positioning algorithm is presented in [34] to address the absorption effects of crowded scenarios on Wi-Fi signals through an indoor canyon environment and crowed sensing is achieved by using a mobile camera with the assistance of deep Convolutional Neural Network (CNN). A similar approach of location estimation using the merging of multiple heat-maps is discussed in the Probability Maps Technique [35]. The probability map technique uses RF packet transmission to asses RSSI Value of a transmitter mobile node at multiple receivers. This technique differs from our proposed technique in such a manner that it uses probability distribution for distance calculation and uses a triangular density function and geometric correction technique in the localization procedure. An online path-loss parameter estimation approach is discussed in [36] and a variant of particle filter (RBPF-AGB) is used for position estimation of target node. In [37], the author has proposed handling LOS and NLOS cases separately using the corresponding path loss model to enhance indoor WiFi positioning accuracy.

Although most of the proposed systems avoid a pre-offline survey process, the crowd-sourcing activity requires considerable time for active or inactive user participation; additional sensors are needed to construct the database, or the environment map adds more complexity in calibration steps. As per our knowledge, the map and AP information are inevitable for location accuracy because the indoor architecture shapes radio propagation; therefore, to achieve a simple IPS and a certain level of accuracy, the map information should be considered. Triangular interpolation and extrapolation (TIX) [18] provides a 4–5 m accuracy without map information, whereas the signal-distance map (SDM) [19] working on the same principle improves the accuracy and is dependent on the location and number of APs in the environment.

## 3. Proposed Approach

As the indoor architecture of the environment shapes the RSSI heat map, the map information cannot be ignored. Maps are easily available in legally approved buildings. The map used in our approach is simple and can be constructed using a measuring tape and a simple bitmap drawing application such as “Microsoft Paint” (Microsoft Inc., Redmond, WA, USA). The other options include the use of automated maps generated from the 2D simultaneous localization and mapping (SLAM) [38]. Our approach consists of the following tasks: generation of a pixel map from the floor plan of the target site, where indoor positioning is required, the generation of an RSSI map from the pixel map using LOS and NLOS models for each AP, and finally, the estimation of the user location by applying the Overlap technique on generated RSSI maps of APs listed in RSSI vectors received at the target location. To complete the entire estimation task, we require three types of information: the bitmap (M×N pixels) of building floor plane (at reasonable resolution, i.e., not very high to avoid image processing computation and not very low to preserve that environment architecture, e.g., 10 pixels/m), i.e., Equation (1); the list of “*k*” Wi-Fi APs installed, location on the map, and the Media Access Control(MAC) address. $V_{AP}\;=\;\left\{AP_{1},AP_{2},...AP_{n}...,AP_{k}\right\}$, where $AP_{n}\;=\;\langle MAC_{n},x_{n},y_{n}\rangle ,\;n\;=\;1,2,3...k,x\;=\;1,2,...,M\;$ and y=1,2,...,N; the received RSSI list $S_{RSSI}==\left\{S_{1},S_{2},......,S_{n},\right\}$, where $\;S_{i}=\langle MAC_{i},l\rangle$ and n=1,2,3... at the target location which includes the Mac address “$MAC_{i}$” and the RSSI level “*l*” of each AP in the range. For convenience, we have considered only *x* and *y* coordinates to address common average floor height buildings where the *z*-axis has no significant impact on RSSI calculation; however, in the case of high roof buildings, e.g., shopping malls, airports, etc., and the floor plan remains the same from floor to ceiling, the mounting height (*z* coordinate) of AP must be considered in RSSI estimation, as RSSI estimation depends on Euclidean distance from source to receiver. Otherwise, the proposed approach requires a 3D model of the environment, which is not commonly available, although it can be built easily using 3D modeling tools. We have described each task as follows.

### 3.1. Pixel Map Tracing

**Figure 2:**
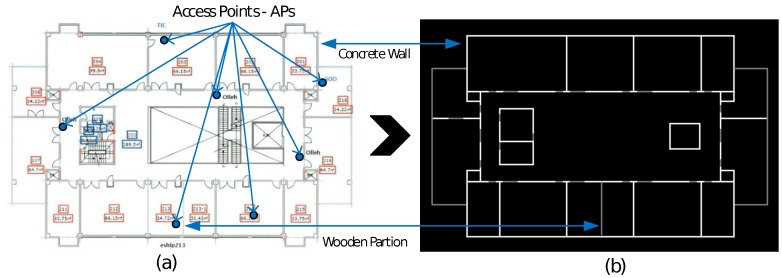
Input: (**a**) detailed floor plan of the Regional Innovation Center (RIC Building) and (**b**) corresponding output: Wall-map obtained from the pixel map tracing process.

As mentioned earlier, the key component of our approach is the pixel map. In our experiment, we have created pixel maps from the floor plans of the subject buildings. This task includes the tracing of a picture and can be easily done using any image drawing tool. First, we resize the floor plan image to a resolution of 10 pixel/m. Then, we trace each concrete wall with white colored (RGB 255,255,255) lines and the wooden partitions with gray colored (RGB 128,128,128) lines on a black background (RGB 0, 0, 0). For simplicity, we consider only two types of walls; the color value is an adjustment factor for the wall attenuation factor (WAF) [13]. The adjustment factor must be carefully selected with respect to the thickness and material of the walls or obstructions. Finally, we obtain a gray-scale image map, Equation (1), with the information of the wall sizes, types, orientation, and location. Figure 2 shows the inputs and output of this pixel map generation task:

(1) $$B\left(M,N\right)=\left[\begin{array}{cccc} px(1,1) & px(1,2) & \cdots & px(1,N)\\ px(2,1) & \ddots & & \vdots \\ \vdots & & px(i,j) & \vdots \\ px(M,1) & \cdots & \cdots & px(M,N) \end{array}\right]$$

where $\;0\leq px_{\left(i,j\right)}\;\leq \;255,i=1,2,3,...,M$ and j=1,2,3,...,N.

### 3.2. RSSI Map Generation

**Figure 3:**
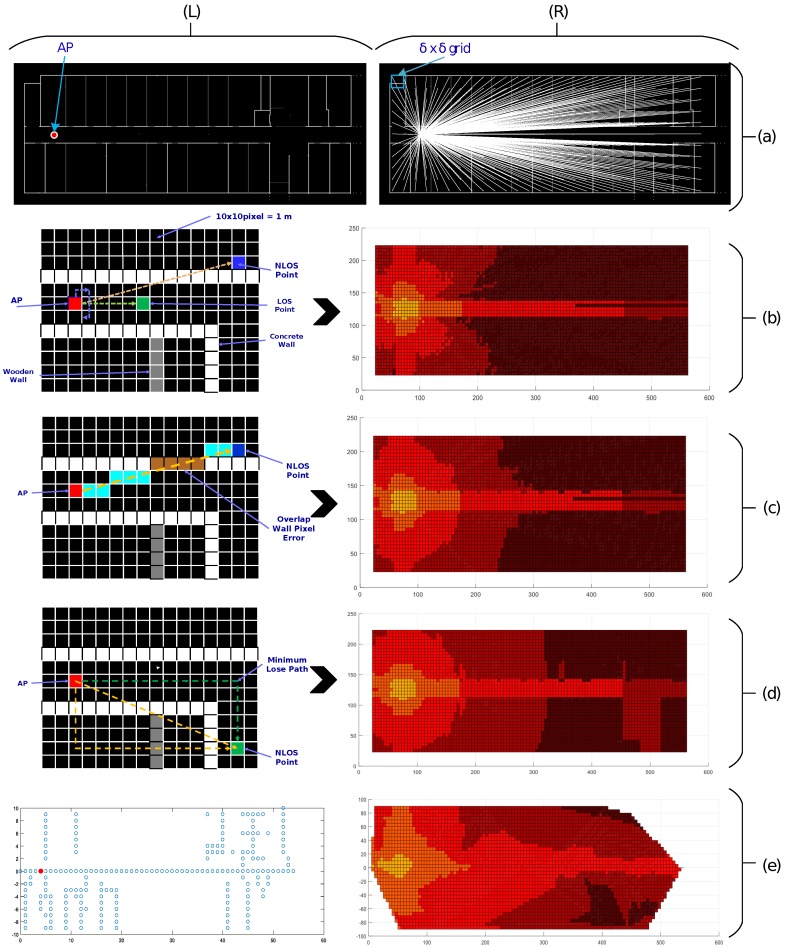
Details of 2nd the task: Generation of the path loss radio-map for an AP and corrections made to improve the estimation of RSSI at particular point on the map. (**a**) Left: IT Building’s traced wall-map; Right: Vector from each grid point to an AP (for clarity, we used a 20 pixel/m resolution) (**b**) Left: Two types of profile vectors from an LOS Point and an NLOS point; Right: Flower-shaped radio map obtained without any correction; (**c**) Left: Pixel overlap of the profile vector with the wall; Right: Radio-map after the 1st correction (**d**) Left: Two rectangular paths, one direct from AP to the estimation point; Right: Radio-map after the 2nd correction and; (**e**) Left: Reference points to measure the actual RSSI map; Right: Real RSSI map of the environment obtained after linear interpolation.

The second important task is to generate the RSSI Map of each selected AP for location estimation. To build an RSSI radio-map, we first divide the map into a grid of equal square blocks of 10 pixels/m. Next, we select a pixel vector from the center of a block (let (*p*,*q*)) to the AP (let (*x*,*y*)) using an improfile function that returns the intensity values of pixels along a straight line, i.e., $V\left(\left(p,q\right),\left(x,y\right)\right)\;=\;improfile\left(\left(p,q\right),\left(x,y\right)\right)\;=\;\left\{px(p,q),px(p\pm 1,q\pm 1),........,px(x,y)\right\},$ where px(p,q) returns intensity values of pixels at location (p,q),p=δ, 2δ, 3δ, ..., *M*,q=δ, 2δ, 3δ, ...,*N* and delta is the size of grid spacing, as shown in Figure 3a-(right). We call this pixel vector a profile vector. This profile vector enables us to differentiate between the LOS and NLOS scenarios, in order to determine the type and number of walls or obstructions in the path, and the length of the path as well. If all of the pixels in the profile vector are black, it indicates an LOS case; otherwise, it is NLOS. The white pixels in the profile vector indicate the number of walls in the path from the center of the block to the AP. Equation (2) describes the calculation of the path loss weight (PLW) from the profile vector by finding the peaks values in the pixel array of the profile vector using *GetPeakValues*() that return local maxima and their count in given vectors. Next, the product of the PLW and WAF calculates the effective path loss (EPL), Equation (3), for NLOS radio signal propagation, where PLW represents the adjustment factor for walls in paths. The number of pixels in the profile vector is the absolute distance between the center of the reference block and the AP; however, to avoid diagonal error, we measure the Euclidean distance from the center of the block to the AP, as in Equation (4). Using this information and the RSSI prediction model, Equation (5), we calculate the RSSI for a particular block of the LOS or NLOS case.

(2) $$PLW=\frac{\sum GetPeakValues(V\left((p,q),(x,y)\right))}{255},$$

(3) EPL=PLW×WAF,

(4) $$d_{\left((p,q),(x,y)\right)}=\sqrt{{\left(p-x\right)}^{2}+{\left(q-y\right)}^{2}},$$

(5) $$f\left(p,q\right)=p_{0}+10\times r\times log\left(\frac{d_{\left((p,q),(x,y)\right)}}{10}\right)+EPL,$$

where WAF = 3.5, $p_{0}$ = −30 dB and $r\;=\;\left\{\begin{array}{cc} 1, & PLW=0,\\ 1.6, & PLW>0, \end{array}\right.$

(6) $$M\left(AP_{k}\right)=\left[\begin{array}{cccc} f(\delta ,\delta ) & f(\delta ,2\delta ) & \cdots & f(\delta ,n)\\ f(2\delta ,\delta ) & \ddots & & \vdots \\ \vdots \\ f(m,\delta ) & \cdots & \cdots & f(m,n) \end{array}\right]$$

where m=M/δ,n=N/δ. To generate the RSSI path loss map, we repeat this process for all blocks and we get a M(m,n) matrix Equation (6) of the RSSI map of current AP. Algorithm 1 shows implementation details of Map Generation task.

Similarly, we generate an RSSI Map for each AP and store them in a list, i.e., $E_{M}\;=\;\left\{M_{S_{1}},M_{S_{2}},M_{S_{3}},...,M_{S_{i}},...,M_{S_{k}}\right\}$, where i=1,2,3...k, and each $M_{S_{i}}$ is a tuple of $\langle mac_{i},M_{i},loc_{i}\rangle \;=\;\langle Mac\;Address,Estimated\;radio\;map,\;Location\;of\;AP\;on\;bitmap\rangle$. The RSSI map of each AP is tagged with its MAC address for later reference in position estimation procedure. A mesh plot of an estimated RSSI Map is shown in Figure 1.

#### RSSI Estimation Model

**Figure 4:**
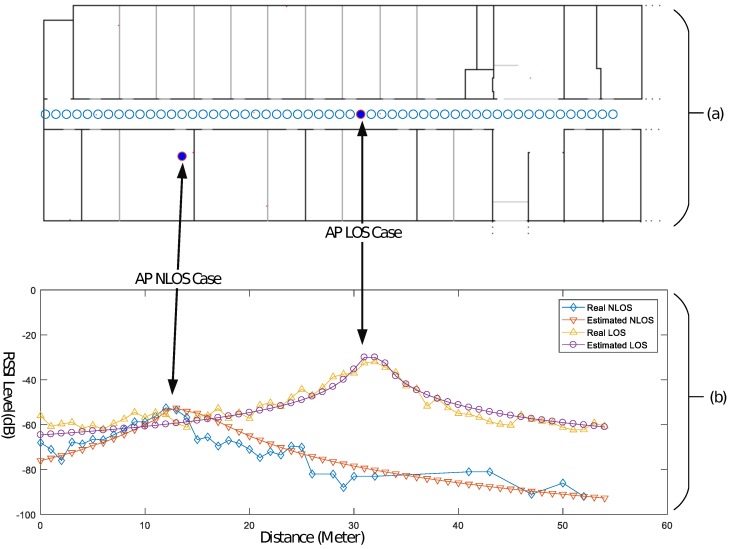
(**a**) reference points to measure actual RSSI level received by a handheld device inside an office environment (IT Building) and (**b**) real and estimated RSSI with respect to distance for both LOS AP and NLOS AP cases.

The location accuracy of our approach is completely dependent on the correctness of the RSSI map, whereas the correctness of the RSSI map depends on the accuracy of the RSSI prediction model selected for each point on the map. We have analyzed multiple RSSI path loss models and their accuracy with the respect to LOS and NLOS cases and selected soft partition and the concrete wall attenuation factor model [13,39]. Figure 4 depicts the estimated and the real RSSI levels received, with respect to the distance, for the selected models. During the assessment of the RSSI path loss using the wall-map approach, we found that a couple of corrections were required in the estimation, as described in the following sections.

#### Correction I

Pixel line errors occur when the inclination angle of the profile vector with any wall in the map is very small, i.e., multiple pixels overlap over the wall, as shown in Figure 3c-(left). As the pixels in the profile vector indicate free space and the thickness of the obstruction, due to this overlap, an error is caused and the RSSI map appears more like a petal shape around the walls, as displayed in Figure 3b-(right). To mitigate the affect of the pixel line error, we count the peaks’ values in the path vector. The output of correction I is shown in Figure 3c-(right).

#### Correction II

To improve the RSSI map generation accuracy using the procedure described above, we compare our map with a real RSSI heat map, calculated manually by site survey, while recording the RSSI information at each crossing point, 1 m in the horizontal and vertical direction of the grid, as depicted in Figure 3d-(left). We found that the original heat map shows a corner diversion due to the dominant path [40], whereas in our RSSI map estimation, only the direct path was used. To incorporate such effects and to retain a low calculation complexity, we add two triangular paths to avoid the direct path error to a certain extent, as shown in Figure 3d-(right). Finally, we select a path with a minimum path loss, calculated by following each of the three paths. This correction is more useful if the building walking paths are rectangular and aligned with the map’s *x*- and *y*-axis.

**Algorithm 1** Generate RSSI Map1:**procedure** GenRSSIMap2:***INPUT***:3:    Map←PathofMapfile.bmp4:    APLoc←LocationofAPonMap(x,y)5:    Delta←GridBlockwidthδ6:***OUTPUT***:7:    RSSIMap←RSSIMapofAPatlocationAPLoc8:***BODY***:9:    Height←HeightofMap10:    Width←WidthofMap11:    GPoint←Get_Grid_Points(Delta,Width,Height)12:***loop***: Estimate RSSI for each point ’px’ in ’GPoint’13:    $\mathrm{Vp}0\leftarrow \mathrm{GetProfileVector}(\mathrm{map},[{\mathrm{px}}_{x},{\mathrm{APLoc}}_{x}],[{\mathrm{px}}_{y},{\mathrm{APLoc}}_{y}])$14:    **if** lengthof(Vp0)=Delta **then**15:        ${PeakValues}_{Vp0}\leftarrow findpeaks\left(Vp0\right)$16:        **if** Vp0isnotstartingatwall **then**17:           $x1\leftarrow [{\mathrm{px}}_{x},{\mathrm{px}}_{x},{\mathrm{APLoc}}_{x}]$18:           $y1\leftarrow [{\mathrm{px}}_{x},{\mathrm{APLoc}}_{y},{\mathrm{APLoc}}_{y}]$19:           Vp1←GetProfileVector(map,x1,y1)20:           ${PeakValues}_{Vp1}=findpeaks\left(Vp1\right)$21:           $x2\leftarrow [{\mathrm{px}}_{y},{\mathrm{APLoc}}_{x},{\mathrm{APLoc}}_{x}]$22:           $y2\leftarrow [{\mathrm{px}}_{y},{\mathrm{px}}_{y},{\mathrm{APLoc}}_{y}]$23:           Vp2←GetProfileVector(map,x2,y2)24:           ${PeakValues}_{Vp2}\leftarrow findpeaks\left(Vp2\right)$25:           $PLW\leftarrow \min(\sum \left({PeakValues}_{Vp0}\right),\sum \left({PeakValues}_{Vp1}\right),\sum ({PeakValues}_{Vp3})$26:        **else**Calculatedirectpath27:           $PLW\leftarrow \sum ({PeakValues}_{Vp0})$28:    **else**29:        PLW←030:    p0←−3031:    EPL←032:    WAF←3.533:    r←1;34:    **if** PLW>0 **then**35:        r←1.6;36:        EPL←PLW/255×WAF;37:    $di\leftarrow \sqrt{(}{\left({\mathrm{px}}_{x}-{\mathrm{APLoc}}_{x}\right)}^{2}+{\left({\mathrm{px}}_{y}-{\mathrm{APLoc}}_{y}\right)}^{2})$38:    estRSSI←(p0−10×r×log(di/10)−EPL)39:    $RSSIMap({\mathrm{px}}_{x},{\mathrm{px}}_{y})\leftarrow estRSSI$40:    **goto** *loop*.

### 3.3. User Location Estimation

The LOCALI location estimation is also an easy and interesting process. As an indoor environment is nonlinear, we cannot estimate the location using trilateration. This nonlinearity causes non-circular RSSI regions around the transmitter, and it is not possible to obtain an accurate estimation using the time or RSSI information directly. To estimate the user location, we propose a four-step procedure that includes selection, thresholding, intersection, and centroid finding.

**Figure 5:**
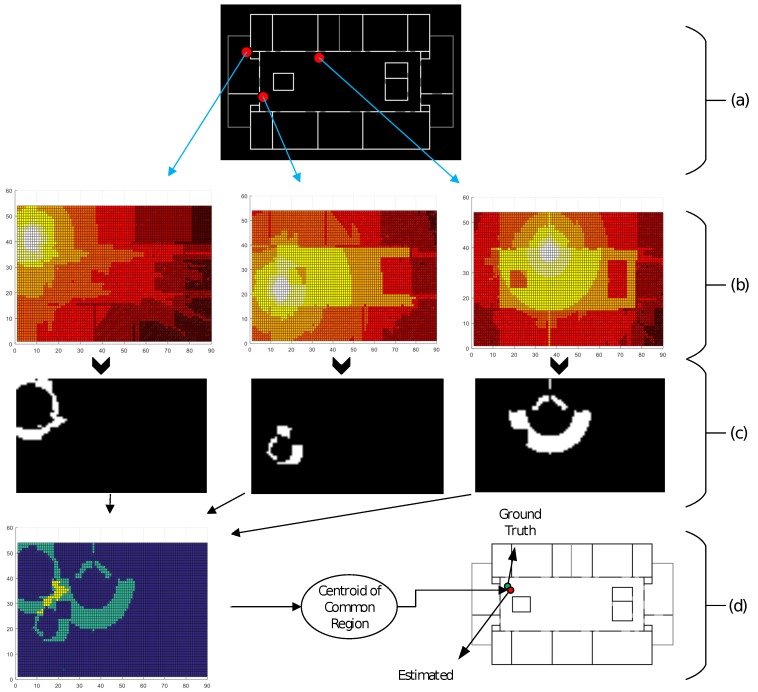
Details of the 3rd task for target location estimation: (**a**) location of the selected APs with the strongest RSSI received by the target/user, on the map; (**b**) radio-map of each selected AP; (**c**) binary map obtained after applying the threshold value, depicting the expected region around an AP, where the target is located; and (**d**) intersection of the binary maps and location, considering the centroid of the common region.

- Selection of APs for location estimation; for simplicity, we select the top three APs in the list with the strongest RSSI level.
- For each AP, we generate a binary map by applying the threshold value of the RSSI level received from a particular AP on the corresponding RSSI Map. Let us assume that we have received a list $Z=\left(-56,mac_{A}\right),\left(-70,mac_{B}\right),\left(-65,mac_{C}\right)$. Each RSSI Map is an m×n matrix of RSSI levels and we mark a value of “1”, if the block has a 3 dB-difference value with the RSSI level received at the target, else it is “0”. This process gives us a binary map, as in Figure 5c. The same process applies for the remaining two selected APs, for the assessment of the RSSI map.
- We now have three binary maps that depict the expected region around each AP, where a target can be located. Next, we perform a simple intersection operation between the three binary maps and obtain the common region for these maps; this gives us a region, where all the points have the same values, for the three selected APs, from the received RSSI list “*Z*”, at the target location.
- Finally, the centroid of the common area gives us the estimated location of the target. The step-by-step implementation details of this task are listed in Algorithm 2.

**Algorithm 2** Estimate Target Location1:**procedure** GetTargetLoc2:***INPUT***:3:    $RSSIMaps\leftarrow \mathrm{List}\;\mathrm{of}\;\mathrm{Maps}\;“E_{M}”$4:    $S_{RSSI}\leftarrow \mathrm{List}\;\mathrm{of}\;\mathrm{RSSI}\;\mathrm{Values}<(\mathrm{RSSI}\;\mathrm{Level},\;\mathrm{MAC})>\;\mathrm{Received}\;\mathrm{by}\;\mathrm{Target}\;$5:***OUTPUT***:6:    $TargetLoc\leftarrow (t_{x},t_{y})\;\mathrm{Location}\;\mathrm{of}\;\mathrm{Target}$7:***BODY***:8:    $APList\leftarrow \mathrm{Get}\;\mathrm{Top}\;\mathrm{Three}\;\mathrm{APs}\;\mathrm{with}\;\mathrm{Highest}\;\mathrm{RSSI}\;\mathrm{Level}\;\mathrm{from}\;S_{RSSI}$9:    ORMap←RSSIMaps[0]×010:***loop***: Threshold RSSIMap for each AP in APList11:    map←GetRSSIMapOf(AP.MAC,RSSIMaps)12:    level←AP.RSSILevel13:    thMap←(map−level)14:    binMap←(abs(thMap)<3)15:    ORMap←ORMap|(binMap)16:    **goto** *loop*.17:    $comRegion\leftarrow GetRegion(ORMap{,}^{\prime }Centroid^{\prime })$18:    TargetLoc←comRegion.Centroid

## 4. Experiment Setup

We have selected two types of environments for the experimentation and verification of our proposed approach: a hallway environment of 2160 m${}^{2}$ and an atrium building 1215 m${}^{2}$, at the Yeungnam University, the 2nd floor of an IT Building and the 1st floor of an RIC Building, respectively. Figure 2a and Figure 4a show the floor plans of both the sites, respectively. The setting up of the LOCALI-based ILBS is a two-step process: the first is the creation of a map file server by calculating the RSSI map of each Wi-Fi AP permanently installed at the site. Next is the location estimation, using the overlap algorithm. The server is a simple Personal Computer (PC) with Intel Corei3 Processor and 8-GB RAM, running MATLAB (16a, MathWorks, Inc., Natek, MA, USA). We performed all the experiments in the hallway of an IT Building for calculating the parameters, “r” and “WAF”, and used the RIC Building for testing. The IT Building contains 21 APs and only 12 are selected, whereas, from the RIC Building, only seven are selected to participate in the position. To estimate the RSSI map for each selected AP, we selected a grid with delta = 5 instead of delta = 10, for radio-maps with slightly high resolution. To measure the positioning accuracy of the proposed technique, 54 reference points 1 m apart from each other are selected in the IT Building and 98 reference points are selected in a rectangular path around the opening between different floors of the RIC Building. Android and Apple-based hand-held devices are used for the RSSI calculation and as the target devices at each reference point marked on floor.

## 5. Results and Discussion

**Figure 6:**
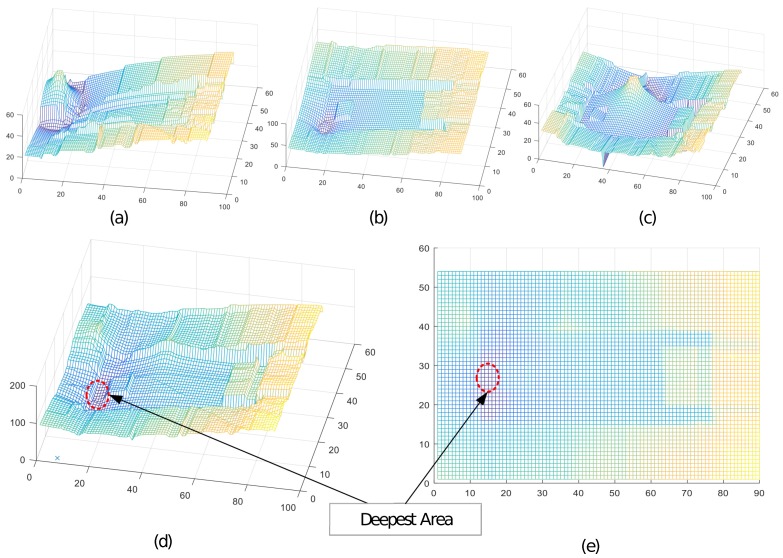
Slightly different approach from the binary map for reducing the thresholding calculation: (**a**) expected target location of the lowest valley with respect to AP, A; (**b**) expected target location of the lowest valley with respect to AP, B; and (**c**) for AP, C; (**d**,**e**) combination of the RSSI valleys and deepest region of the expected target location.

To estimate the position on each reference point on the map, instead of generating binary maps in the thresholding process, we consider absolute values at each grid point of the map, after subtracting the corresponding RSSI level received at the target for a particular AP. This gives us a mesh grid with a circular valley showing the expected location of the target around each AP, as shown in Figure 6a–c. Subsequently, we add all three of the maps and determine the deepest location in the combination map, as depicted in Figure 6d,e. This deepest location indicates the estimated location of the target. The difference between ground truth and estimated location are calculated by Euclidean distance between corresponding points on the bitmap.

**Figure 7:**
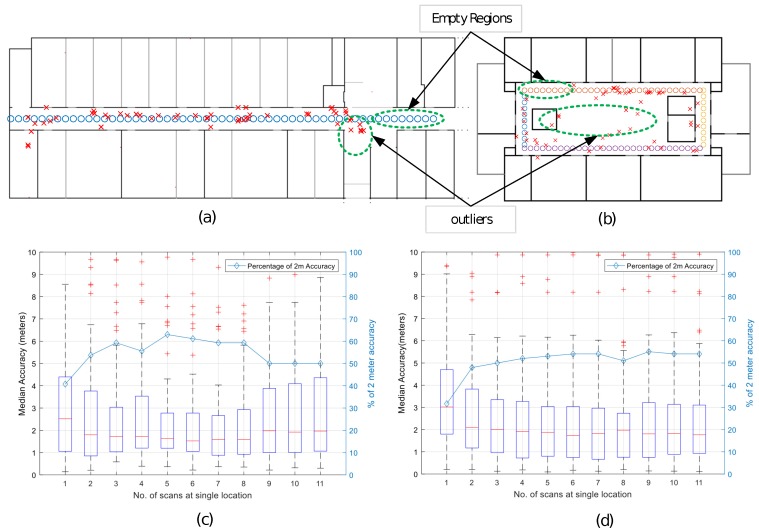
(**a**) ground truth reference points of the RSSI received from user “circles” from one end of the corridor to the other and localization points of the user “Xs” estimated by the system, along a straight path of the IT Building; and (**b**) actual reference points of the user and estimated localized points around the open area of the RIC Building hallway; (**c**,**d**) median accuracy in meters and percentage less than 2 m accuracy with respect to number of scans taken at a reference point for the IT and RIC Buildings, respectively.

In Table 1, we have compared our approach with some other similar or slightly similar model-based approaches. As the table shows, our approach is more practical and adoptable in nature, as it does not requires any active user input and special purpose hardware resources. The automatic assessment of LOS and NLOS and obstacle count (walls) is special to our approach, whereas some techniques employ a single model for LOS/NLOS cases. In addition, our technique does not use interpolation and gives more precise changes between LOS and NOLS cases. Active user input in [34] and hardware requirements of [35] make them less practical techniques due to limited mobile resources and user point of view. Moreover, Table 2 presents statistics regarding the time and memory consumption in our setup. The results demonstrate that our proposed technique sets up the IPS without a time-consuming effort and is ready to use within a few minutes. No significant resources in terms of the memory are required. The results in Figure 7 show the percentage of 2 m position accuracy achieved by the system, without the application of any optimization technique.

In the proposed technique, there are several aspects for improving the accuracy of the RSSI map estimation that are critical and need further exploration; e.g., the selection of the AP for position estimation, the location estimation procedure, and the optimization methods for enhancing the location accuracy. In this work, for simplicity, we selected three APs with the strongest RSSI levels as the target locations. The architecture of the environment around an AP also affects the RSSI levels because it is observed that the Wi-Fi signals add up to a stronger RSSI at the closed-end of the hallway. This effect causes certain nonlinear behavior in the real-time RSSI value for the LOS environment, compared to the estimated RSSI value and results as an error in position estimation at the close ends. Furthermore, the APs closer to the wall show stronger signals on opposite sides due to reflection of rays towards free space and cause free-space outliers, as highlighted in Figure 7. These effects are observed in closed-end corridors and atrium buildings, respectively. As our approach currently does not cover the reflection effect, this limitation can be overcome by placement of the APs at the center of rooms and closer to the ceiling. Moreover, it is observed that the user direction causes an almost 10-dB difference in the measurement at close proximity about three to six meters from transmitters in indoor environments, which is an error of almost 2 m.

## 6. Conclusions

In this study, we have presented a simple and effortless indoor positioning system that does not require any time-consuming calibration survey of the environment. The proposed technique exploits the floor plan information of the environment to build RSSI based path loss maps of a transmitting source more accurately, and these maps further help in estimating position of the targets in the environment by employing a map overlap technique. Results show that the proposed technique has achieved a reasonable accuracy without using any complex procedures and optimization techniques. Moreover, our next focus is to study how to incorporate dynamic changes in RSSI due to changes in environment over time, which will help in calculating more accurate path loss maps. In addition, fusion of inertial sensing data is planned to add more reliable tracking capabilities to the system. Increasing the accuracy of model-based path loss maps will minimize the outliers count, and, ultimately, a high accuracy in position estimation will be achieved while retaining a simple and affordable approach.

## Figures and Tables

**Table 1:** Comparison with related techniques.

	Techniques	Proposed	CBIPA [34]	Probability	Online	PPLM [37]
Props.		Technique		Maps [35]	PLPE [36]	
LOS/NLOS Assessment		Automatic	No	No	No	Manual
Obstacle count		Yes	No	No	No	No
Coverage		Both	Both	LOS	LOS	Case Level
(LOS/NLOS)						(LOS or NLOS)
Positioning		Map	Turbo RSSI	Map	Particle	Trilateration
Algorithm		Overlapping	model	Overlapping	Filter	
Active User Input		No	Yes	No	No	Yes
Interpolation		No	No	No	Yes	No
Special H/W Requirement		No	Yes(Camera)	Yes	No	No

Camera-Based Indoor Positioning Algorithm (CBIPA), Practical Path Loss Model (PPLM), Path Loss Parameter Estimation (PLPE), Line-of-sight (LOS), Non-line-of-sight (NLOS), Received Signal Strength Indicator (RSSI).

**Table 2:** Statistics of the change in the time and memory required on the disk for map generation with respect to the area and number of Access Points.

Building Type	Area (m^2^)	AP Count	delta (*δ*)	Time (s)	Maps File Size (Kb)
Hallway	20 × 54	21	5	1001	630
			10	267	172
Artium	27 × 45	07	5	360	240
			10	103	60
